# Supplementary material for: Fabrication of Temperature Sensors with High-Performance Uniformity through Thermal Annealing
Source: Materials (Basel). 2023 Feb 10;16(4):1491. doi: 10.3390/ma16041491 (PMC9961983; doi:10.3390/ma16041491)
Supplement: Supplementary file 1 [file materials-16-01491-s001.zip › materials-2178306-supplementary.pdf]

Supporting information

Fabrication of Temperature Sensors with High-Performance Uniformity through Thermal Annealing

Hongrui Liu [1,†], Yongchun Li [2,†], Weiji Xie [2], Xinyi Zhou [1], Jishuang Hong [2], Junfeng Liang [1], Yanghui Liu [1,*], Wei Li [2,*] and Hong Wang [1,*]

[1] School of Materials, Shenzhen Campus of Sun Yat-sen University, Shenzhen 518107, China
[2] Department of Anesthesiology, Sun Yat-sen University Cancer Center, State Key Laboratory of Oncology in South China, Guangzhou 510060, China
* Correspondence: liuyanghui@mail.sysu.edu.cn (Y.L.); liw@sysucc.org.cn (W.L.); wanghong3@mail.sysu.edu.cn (H.W.)
† These authors contributed equally to this work.

1. The I-V curves of the Gr-AC temperature sensor at room temperature

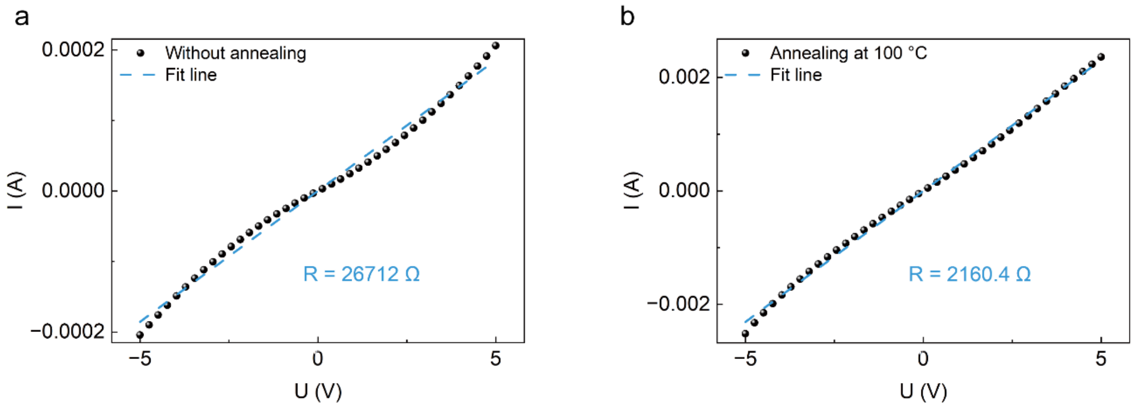

Figure S1. The I-V curves of the Gr-AC temperature sensor at room temperature. After annealing, the DC resistance is significantly reduced.

2. The annealing time

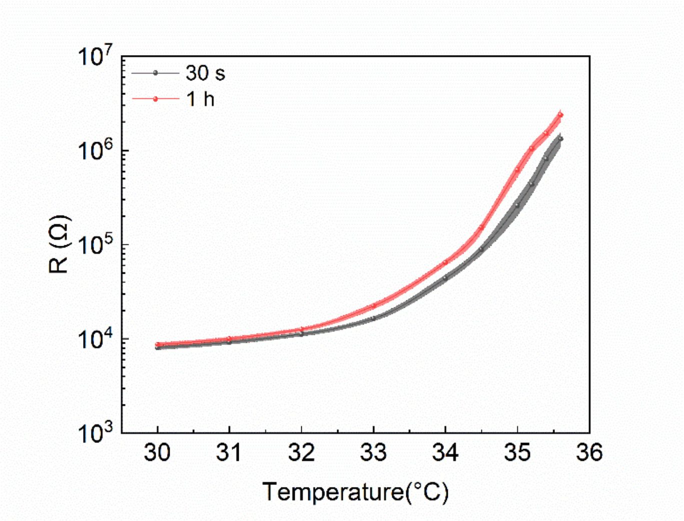

Figure S2. Performance comparison of 10 temperature sensors annealed at 100 °C for 30 s and 1 h. The error bar represents the standard error.

3. Calculation of grayscale values for SEM images

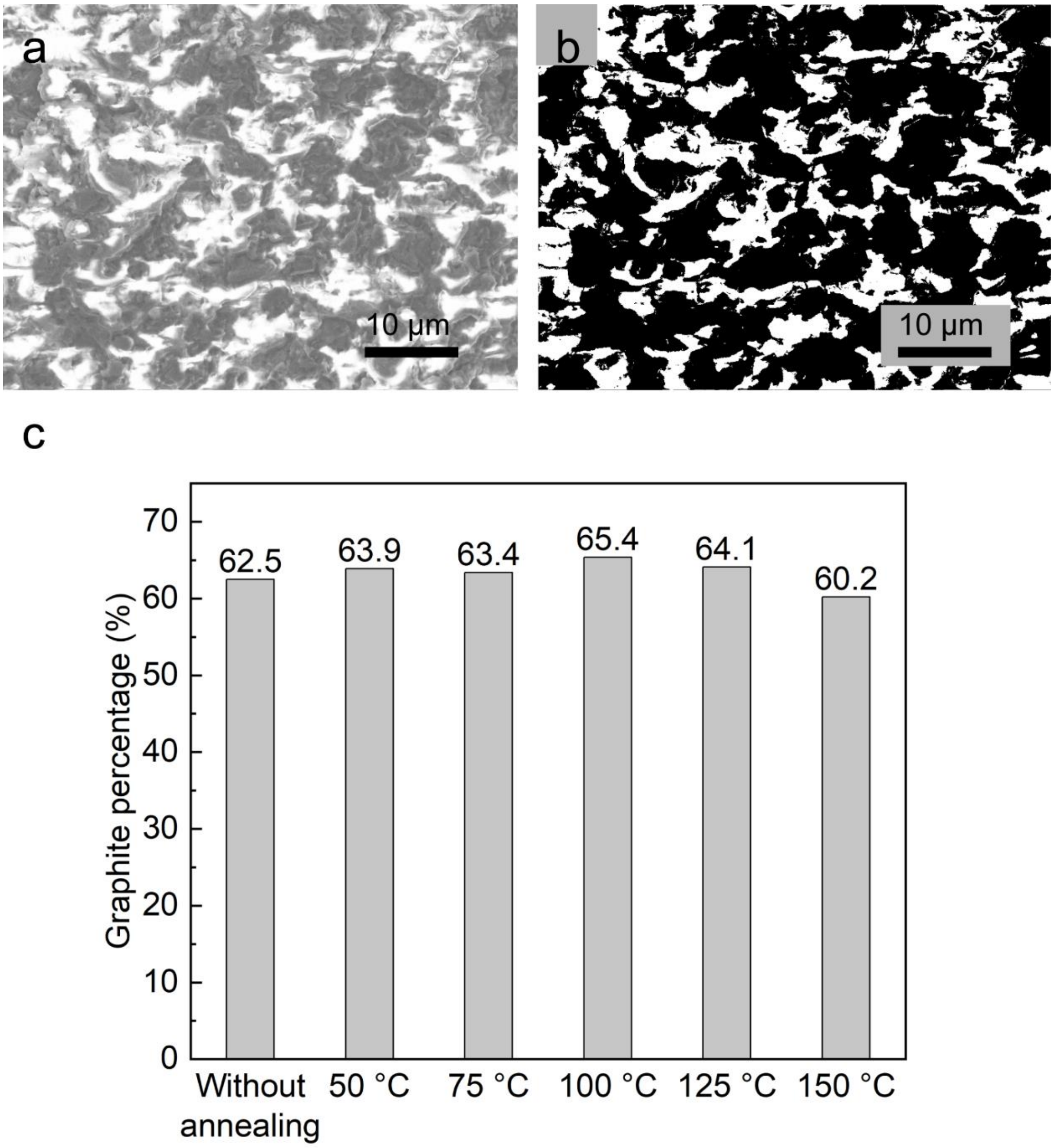

Figure S3. a) The original and SEM image. b) Image after binarization. The grayscale threshold is set to 0.65. c) Graphite percentage of Gr-AC composite under unannealed and annealing at 50, 75, 100, 125, and 150 °C, respectively.
